# *In Vitro* Biodegradation of Resorbable Magnesium Alloys Promising for Implant Development

**DOI:** 10.17691/stm2020.12.6.06

**Published:** 2020-12-28

**Authors:** N.S. Martynenko, N.Y. Anisimova, M.V. Kiselevskiy, D.R. Temralieva, G.I. Raab, E.A. Kornyushenkov, M.V. Rodionov, S.V. Dobatkin, Y.Z. Estrin

**Affiliations:** Researcher, Laboratory of Non-Ferrous and Light Metals; A.A. Baikov Institute of Metallurgy and Materials Science, Russian Academy of Sciences, 49 Leninsky Prospect, Moscow, 119334, Russia; Engineer, Laboratory of Hybrid Nanostructured Materials; National University of Science and Technology “MISIS”, 4 Leninsky Prospect, Moscow, 119049, Russia;; Leading Researcher, Laboratory of Cell Immunity; N.N. Blokhin National Medical Research Center of Oncology, Ministry of Health of the Russian Federation, 24 Kashirskoye Shosse, Moscow, 115478, Russia;; Professor, Head of the Laboratory of Cell Immunity; N.N. Blokhin National Medical Research Center of Oncology, Ministry of Health of the Russian Federation, 24 Kashirskoye Shosse, Moscow, 115478, Russia;; Junior Researcher, Laboratory of Non-Ferrous and Light Metals; A.A. Baikov Institute of Metallurgy and Materials Science, Russian Academy of Sciences, 49 Leninsky Prospect, Moscow, 119334, Russia; PhD Student, Laboratory of Hybrid Nanostructured Materials; National University of Science and Technology “MISIS”, 4 Leninsky Prospect, Moscow, 119049, Russia;; Head of the Laboratory “Technologies of Severe Plastic Deformation (SPD)”; Ufa State Aviation Technical University, 12 K. Max St., Ufa, Republic of Bashkortostan, 450008, Russia;; Head of Experimental Therapy Clinic; N.N. Blokhin National Medical Research Center of Oncology, Ministry of Health of the Russian Federation, 24 Kashirskoye Shosse, Moscow, 115478, Russia;; Senior Researcher, Experimental Therapy Clinic; N.N. Blokhin National Medical Research Center of Oncology, Ministry of Health of the Russian Federation, 24 Kashirskoye Shosse, Moscow, 115478, Russia;; Head of the Laboratory of Non-Ferrous and Light Metals Science; A.A. Baikov Institute of Metallurgy and Materials Science, Russian Academy of Sciences, 49 Leninsky Prospect, Moscow, 119334, Russia; Professor, Department of Metallography and Physics of Strength; National University of Science and Technology “MISIS”, 4 Leninsky Prospect, Moscow, 119049, Russia;; Honorary Professorial Fellow, Department of Materials Science and Engineering; Monash University, Department of Materials Science and Engineering, Clayton, VIC 3800, Australia; Adjunct Professor in the School of Mechanical and Chemical Engineering The University of Western Australia, Department of Mechanical Engineering, Crawley, WA 6009, Australia

**Keywords:** magnesium alloys, biodegradation, implants, ultrafine-grained structure, equal channel angular pressing, cell adhesion, colonization

## Abstract

**The aim of the investigation** was to study the biodegradation characteristics and rate of magnesium alloys *in vitro*.

**Materials and Methods.:**

We studied the biodegradation of magnesium alloys Mg-Zn-Ca and WE43 (Mg-Y-Nd-Zr) in homogenized (initial) condition and after strengthening by mechanical processing using equal channel angular pressing (ECAP). The samples were incubated in a model system based on reference fetal calf serum (FCS) in the static and dynamic modes. The morphology of alloy surfaces was analyzed using light microscopy and computed tomography. Biodegradation was assessed by calculating weight loss within a certain incubation period. Cell adhesion and colonization stimulation were quantified in terms of a cell index (CI) using an analyzer xCELLigence RTCA Systems (ACEA Biosciences, Inc., USA) during the incubation of HEK 293 cells on WE43 specimens.

**Results.:**

Strengthening of magnesium alloys Mg-Zn-Ca and WE43 using ECAP and, consequently, the changed structure resulted in the biodegradation acceleration as high as eightfold. Among the specimens incubated in FCS in different modes, those incubated in liquid flow exhibited the biodegradation rate twice as high as that of the specimens tested under static conditions. The biodegradation process was accompanied by local corrosion, although the degradation was primarily concentrated along the specimen margins stimulating cell adhesion and colonization. Such nature of degradation, as a rule, does not lead to anisotropy of the strength characteristics, that is important for medical materials. Superficial degradation of the alloys with no X-ray density changes in the bulk of the specimens was confirmed by computed tomography.

**Conclusion.:**

The study of the biodegradation rate and further characteristics of magnesium alloys Mg-Zn-Ca and WE43 showed that the materials in both structural conditions are suitable for implants and can be used in bone implants and surgical fasteners.

## Introduction

One of the challenging problems of modern orthopaedics and traumatology is the search for biodegradable implant materials that would undergo biodegradation in the patient’s body when the damaged bone tissue is being remodeled. It would eliminate the need for a repeated surgery for implant extraction [1–4]. Metallic materials, which enable the production of submerged biodegradable implants and fastening tools with desired strength, are of particular interest. Magnesium alloys are among the most promising materials for such applications. They exhibit acceptable biocompatibility and their mechanical characteristics are compatible with those of the cortical bone [5–6]. However, magnesium is known to have an excessively high biodegradation rate in aqueous media. Biodegradation is accompanied by the intensive release of hydrogen, which can have an adverse effect on the activity of cells participating in regenerative processes [7–8].

A great deal of studies has been devoted to the degradation of medical magnesium alloys [9–11]. The most common ones are immersion testing in different media [12], potentiodynamic measurements [13], analysis of the gas evolution rate [14], etc. Most frequently, the tests are performed in a stationary medium that is not consistent with the real functioning of the implant in the body. For more adequate modeling of the processes in the body, it is required to test corrosion under fluid flow conditions, i.e. in a dynamic medium. Moreover, magnesium alloys, by nature, are prone to develop pits. Such non-uniform biodegradation with the formation of localized corrosion areas may give rise to anisotropy of mechanical properties, premature loss of operating characteristics, and damage [15–17]. In this regard, in addition to the degradation rate assessment, we consider it reasonable to study the distribution patterns of corrosion, as well as the depth of the pits.

**The aim of the investigation** was to study the biodegradation rate and further characteristics of magnesium alloys in a biological medium when incubated in the static and dynamic modes.

## Materials and Methods

The studied material was a magnesium alloy WE43 (Mg-Y-Nd-Zr) containing rare-earth elements Y and Nd and the Mg-Zn-Ca alloy. Both alloys were analyzed in two states: the initial coarse-grained (homogenized) condition and the strengthened fine-grained one produced by equal channel angular pressing (ECAP)). The processing technology of the materials and their mechanical characteristics were reported in the previous works [18] — the WE43 alloy, and [19] — the Mg-Zn-Ca alloy.

We studied the degradation processes on the specimens of the alloys, which had the shape of one quarter of a cylinder of ~0.5 cm radius, and ~0.14–0.16 cm length. Before testing the specimens were sterilized by being immersed in ethanol (70%) for 18 h, and then dried in aseptic conditions. The specimens were incubated in fetal calf serum (FCS) (HyClon, Thermo Fisher Scientific, Great Britain) imitating the chemical composition of human internal environment at 37°С, in the static and dynamic modes, for 7 and 3 days, respectively. Each type of material was tested using at least 3 specimens.

The static mode involved the incubation of every individual specimen in 2 ml of FCS, with no exposure to atmospheric air. In the dynamic mode, the alloy samples were incubated in a flow-through cell, which was a part of a closed sterile circuit with FCS circulating under the action of a peristaltic pump (Biomark, Inc., USA) at the rate of 3.5 rpm. The unit maintaining the dynamic incubation mode was placed in a CO_2_-incubator (NUAir, USA) throughout the test period. All experiments were carried out in accordance with aseptic and antiseptic regulations using sterile media and consumables. After the experiment the samples were washed and thoroughly dried. A change in weight was determined by weighing the specimens on electronic scales Ohaus PA64 (Ohaus Corporation, USA). The degradation rate was calculated according to the formula:


$$ML=\frac{m_{0}-m_{f}}{m_{0}}\cdot 100\%,$$

where *m*_0_ is the initial weight, and *m_f_* is the final weight in gram.

The degradation of the specimen was studied using a light microscope Axiovert (Carl Zeiss, Germany).

Surface corrosion and internal structure of the specimens were studied using a CT scanner Philips Brilliance 16 (Philips, Netherlands), voltage: 140 kV, current: 30 mA, slice thickness: 0.8 mm. X-ray density was determined according to standardized Hounsfield units (HU).

Alloy specimens colonized by cells were studied *in vitro* using an analyzer xCELLigence RTCA Systems (ACEA Biosciences, Inc., USA) for measuring the cell index (CI), which was proportional to the electrical resistance, in every well of a special microtiter plate. We studied four WE43 specimens in their initial state and after ECAP, in the form of square-shaped plates, 2 mm thick and 4 mm in length. The specimens were sterilized by immersion in 70% ethanol for 4 h followed by placing each sample into a well of an E-plate 16 (ACEA Biosciences, Inc.) containing 200 μl of culture medium RPMI-1640 (PanEco, Russia) by adding 10% FCS (HyClone, Thermo Fisher Scientific), 4 mM L-glutamine (PanEco) and 1% penicillin/streptomycin (PanEco). Then they were incubated in an analyzer for 24 h at 37°С and 5% carbon dioxide to determine the CI values.

The specimens were washed in a culture medium 15 min after incubation. After complete removal of the medium, we took out two specimens of each alloy and applied on their surfaces the cells of the human embryonic kidney (HEK) 293 cell line (N.N. Blokhin National Medical Research Center of Oncology) resuspended in a culture media based on RPMI-1640 at concentration 8.4 **·** 10^6^ cells per ml (20 μl per specimen), followed by 15-minute incubation. Then 200 μl of the culture medium were added carefully in every plate well. The alloy specimens with cells were incubated for 72 h in a CO_2_-incubator. CI was recorded after 24, 48, and 72 h of incubation, in the wells of co-incubation of alloys and cells (CI (alloy + cells)) and in the wells where the alloy specimens were incubated in a cell-free medium (CI alloy). The CI values in the alloy-free wells were considered as controls.

The experiments with cell cultures were approved by the Ethics Committee of N.N. Blokhin National Medical Research Center of Oncology.

**Statistical data processing.** The findings were statistically processed using Statistica 6.0 (StatSoft Inc., USA). Within-group data were checked for normalcy of distribution using Shapiro–Wilk W-test, p>0.05 was the evidence that the distribution under study was not different from normal. Descriptive statistics of every criterion under study within a group was represented as M±SD, where m is the arithmetic mean and SD is the standard deviation. To assess every criterion within a group under study we used three or four specimens of one type of the alloy incubated under similar conditions; the measurement values of each alloy characteristic were considered in triplets. A one-tailed Student’s t-test was used to determine statistical significance of differences of quantitative characters in intergroup comparison of the ECAP-modified alloy state compared to that in the homogenized condition evaluated in duplicate. The differences were considered significant for р≤0.05.

## Results and Discussion

**Figure 1:**
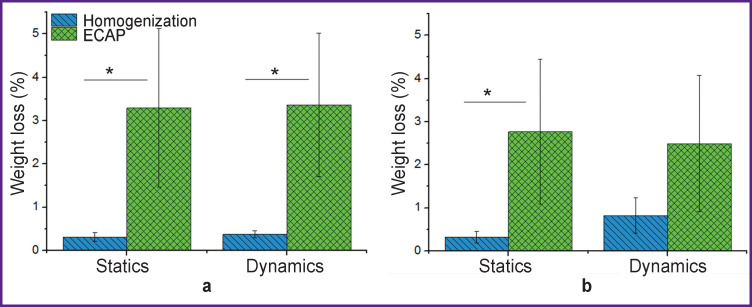
Biodegradation rate of the alloys: (а) Mg-Zn-Ca and (b) WE43 in the static mode and under the fluid flow conditions; * p<0.05

The alloys treated by ECAP showed an increased degradation rate. After the incubation in FCS, the relative weight loss of homogenized samples of Mg-Zn-Ca was 0.31±0.10%, while that of the samples treated by ECAP was 3.29±1.83% (p<0.05; Figure 1 (а)). The weight loss of the WE43 alloy specimens was 0.32±0.14 and 2.77±1.66% in their initial condition and after ECAP, respectively (p<0.05; Figure 1 (b)). The tests conducted under dynamic conditions exhibited similar biodegradation kinetics. The weight loss of the Mg-Zn-Ca alloy in the homogenized condition was 0.37±0.08% and in the ECAP-strengthened condition 3.36±1.66% (p<0.05, see Figure 1 (а)). These parameters for WЕ43 alloy before and after mechanical processing by ECAP were 0.82±0.41% and 2.49±1.58%, respectively (see Figure 1 (b)).

The increase in biodegradation rate of alloys after ECAP is likely to be related to structural changes of the alloys due to the mechanical processing. In our study [19], ECAP of the Mg-Zn-Ca alloy was found to result in the decrease in grain size by a factor of ten. However, the structure of the alloy after deformation was rather non-uniform, which might have caused the acceleration of the bio-corrosion process. It was suggested that the particles of a corrosion-resistant phase Mg_41_Nd_5_ formed in the WE43 alloy could act as micro-cathodes producing numerous micro-corrosion pits on the material surface leading to faster degradation of the less corrosion-resistant matrix acting as an anode [20].

Figure 1 shows that the weight loss values of the alloys studied in both states were nearly the same in the static and dynamic conditions. However, it should be taken into consideration that static testing was conducted for 7 days, while dynamic ones were run over 3 days only. Biodegradation acceleration in dynamic testing can be associated with the possible prevention of the formation of a protective layer of magnesium oxide and hydroxide on the specimen surfaces due to liquid flow [21].

**Figure 2:**
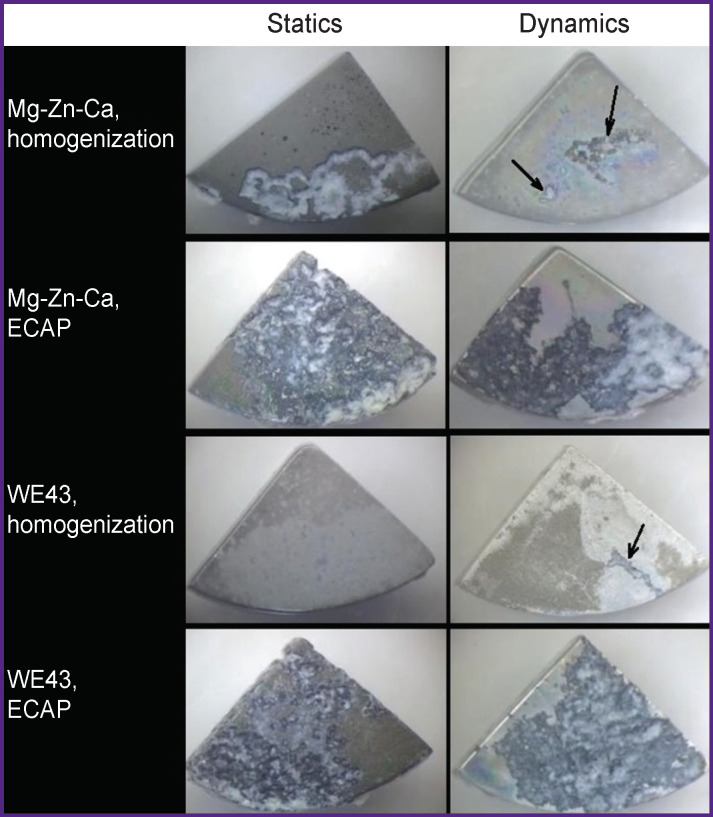
Degradation of Mg-Zn-Ca and WE43 alloy samples after incubation in FCS in the static and dynamic modes The photos are light microscopy images. Arrows show local corrosion of the alloy

After incubation in FCS, the surfaces of both alloys showed non-uniform corrosion distribution. However, the initial WE43 alloy exhibited more uniform biodegradation in both static and dynamic conditions. Furthermore, both alloys after ECAP were characterized by a greater degree of uneven surface damage (Figure 2). The examination of specimens revealed the degradation in all cases to have a peripheral pattern, i.e. being localized primarily at the specimen edges. As the incubation time increased from 3 to 7 days the degradation spread to new surface areas rather than penetrating deep. Such kind of degradation, generally, does not give rise to anisotropy of material strength. This gives promise of potential applicability of magnesium alloys for implant production. Local areas of deep corrosion with singular or multiple pits may cause anisotropy of the mechanical characteristics of the material. Anisotropy of alloy strength can cause premature damage of a prosthetic device (such as an implant or a fastening element) prior to the remodeling of osseous tissue defects. ECAP of magnesium alloys that are otherwise prone to pitting contributes to an increase of the service life of an end product. Improved mechanical characteristics enable an ultrafine-grained alloy to preserve the in-service properties of a prosthetic device for a longer time compared to the homogenized material, as the reduced thickness of the products due to corrosion, while affecting its strength, will be not so dramatic.

**Figure 3:**
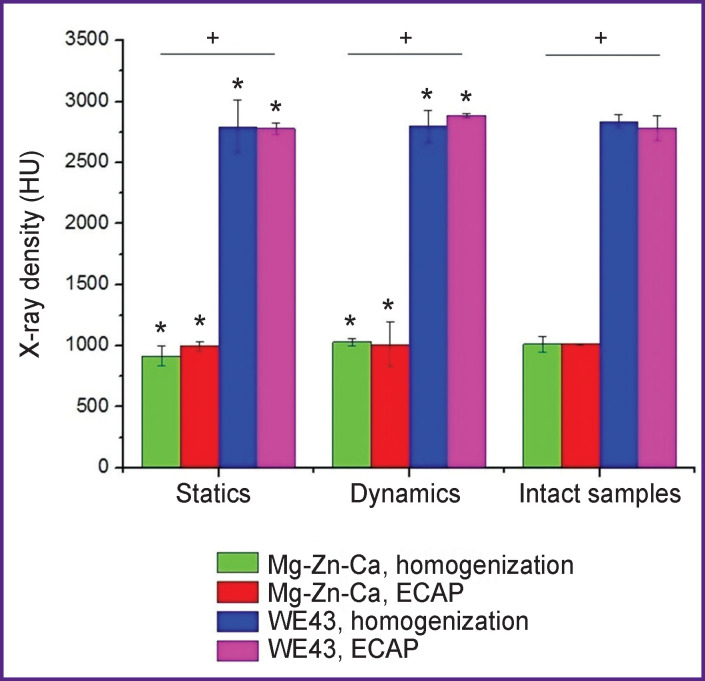
X-ray density of magnesium alloys Mg-Zn-Ca and WE43 before and after the incubation in FCS in the static and dynamic conditions * Significant differences in relation to an initial CI level for intact samples (p<0.05); ^+^ significant differences between the WE43 and Mg-Zn-Ca alloys (p<0.05)

**Figure 4:**
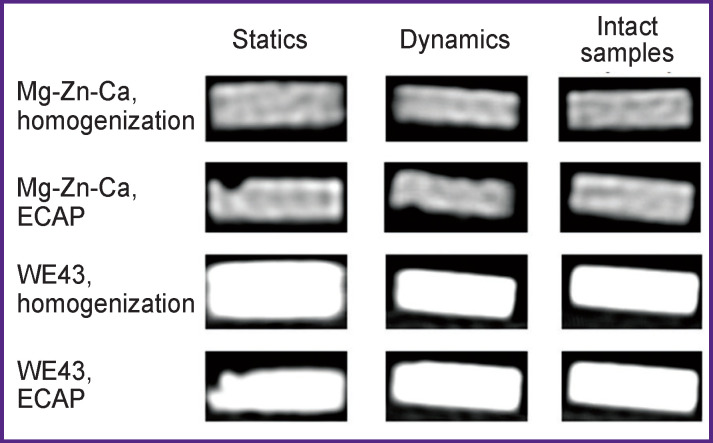
Radiographs of the samples before and after degradation

The peripheral character of degradation representing the changes of the surfaces of the specimens and their total volume was studied based on the assessment of X-ray density (intact specimens) before and after the incubation in FCS (Figures 3 and 4). The incubation of both alloys did not result in altered density, which suggests that no changes occur in the bulk of the material during degradation. In addition, pitting was found in the Mg-Zn-Ca alloy only after ECAP and after the incubation in the static mode.

The differences in the X-ray density of Mg-Zn-Ca and WE43 alloys can partially be due to different values of their mass density (1.75 and 1.84 g/cm^3^ for Mg-Zn-Ca and WE43, respectively).

**Figure 5:**
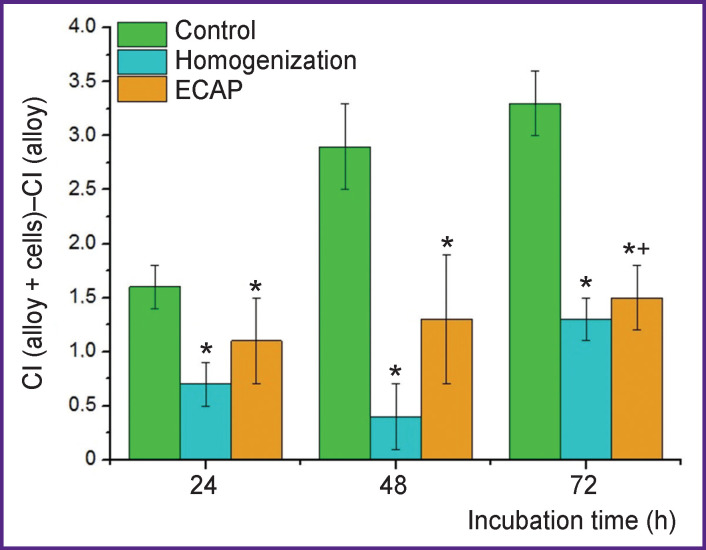
Colonization by cells (HEK 293 line) of WE43 specimen surface in homogenized condition and after ECAP compared to that of the controls (intact cells) The cell index obtained for the wells with the co-incubated cells on the alloys (CI (alloy  +  cells)) versus that in the wells of incubation of similar alloy samples in a cell-free medium (CI (alloy)); * significant differences of the values in relation to the initial CI taken as a control (p<0.05); ^+^ significant differences of WE43 alloys after ECAP compared to WE43 in the homogenized condition (p<0.05)

The experimental results of the incubation of alloy specimens with cell culture *in vitro* showed that the degradation character of magnesium alloys promotes fast cell adhesion to the specimen surfaces followed by their colonization. In particular, a 15-minute co-incubation of cells on the alloys based on WE43 was found to exhibit cell fixation on sample surfaces. The amount of the cells was shown to provide significant differences in CI alloy + cells compared to the CI alloy throughout the experiment (Figure 5). By considering the results presented above, one can assume that cell adhesion is promoted by the peripheral nature of degradation over the 24-hour period of pre-incubation of alloys in a culture medium based on RPMI-1640 before the contact with the cells. The ECAP-processed WE43 alloy stimulated the colonization of cells on the surface more intensively than that in the homogenized condition. After 72-hour incubation the respective values of CI were 1.5±0.3 and 0.4±0.2 (p<0.05), which was likely associated with the accelerated biodegradation that caused the micro-surface of WЕ43 processed by ECAP to have the morphology more favorable for cell colonization than in the case of the homogenized alloy.

It can thus be asserted that the strengthening of magnesium alloys Mg-Zn-Ca and WE43 by ECAP results in a concomitant acceleration of biodegradation. In the dynamic mode imitating the alloy being in a blood flow the biodegradation both in the initial state of the alloys and after ECAP was twice as fast as in the static mode. The degradation of the magnesium alloys studied was accompanied by the formation of local peripheral corrosion areas. Peripheral degradation is not considered to cause anisotropy of the strength characteristics of the alloy [22]. It means that the revealed accelerated biodegradation of ultrafine-grained alloys cannot prevent their use in orthopedics. However, it is our belief that to make the picture complete it is necessary to increase the corrosion testing time to specify the biodegradation characteristics at later incubation stages. Improved strength characteristics compared to homogenized samples indicate that magnesium alloys can be regarded as promising candidates for prosthetic implants. The conclusion is confirmed by the results of the co-incubation of WE43-based alloys with the cells. The results showed preferential stimulation of cell adhesion and colonization of the surface of WE43 specimens after ECAP compared to cellular response of WE43 in the initial condition.

## Conclusion

The presented data on the character and rate of biodegradation of magnesium alloys Mg-Zn-Ca and WE43 (both in the initial homogenized condition and after ECAP employed to produce the ultrafinegrained structure) suggest the prospects for using these materials in bio-resorbable prosthetic implants and fastening elements. The findings of the present study and the previously published research on biocompatibility of magnesium alloys open the way to testing on experimental animals aiming at revealing the nature of their biodegradation *in vivo*.
